# Deciphering the Molecular Targets and Mechanisms of HGWD in the Treatment of Rheumatoid Arthritis via Network Pharmacology and Molecular Docking

**DOI:** 10.1155/2020/7151634

**Published:** 2020-08-26

**Authors:** Wei Liu, Yihua Fan, Chunying Tian, Yue Jin, Shaopeng Du, Ping Zeng, Aihua Wang

**Affiliations:** ^1^Department of Rheumatism and Immunity, First Teaching Hospital of Tianjin University of Traditional Chinese Medicine, Tianjin 300193, China; ^2^Tianjin University of Traditional Chinese Medicine, Tianjin 301617, China; ^3^GuiZhou University of Traditional Chinese Medicine, Guiyang 550025, Guizhou, China

## Abstract

**Background:**

Huangqi Guizhi Wuwu Decoction (HGWD) has been applied in the treatment of joint pain for more than 1000 years in China. Currently, most physicians use HGWD to treat rheumatoid arthritis (RA), and it has proved to have high efficacy. Therefore, it is necessary to explore the potential mechanism of action of HGWD in RA treatment based on network pharmacology and molecular docking methods.

**Methods:**

The active compounds of HGWD were collected, and their targets were identified from the Traditional Chinese Medicine Systems Pharmacology Database (TCMSP) and DrugBank database, respectively. The RA-related targets were retrieved by analyzing the differentially expressed genes between RA patients and healthy individuals. Subsequently, the compound-target network of HGWD was constructed and visualized through Cytoscape 3.8.0 software. Protein-protein interaction (PPI) network was constructed to explore the potential mechanisms of HGWD on RA using the plugin BisoGenet of Cytoscape 3.8.0 software. Gene ontology (GO) analysis and Kyoto Encyclopedia of Genes and Genomes (KEGG) were performed in R software (Bioconductor, clusterProfiler). Afterward, molecular docking was used to analyze the binding force of the top 10 active compounds with target proteins of VCAM1, CTNNB1, and JUN.

**Results:**

Cumulatively, 790 active compounds and 1006 targets of HGWD were identified. A total of 4570 differentially expressed genes of RA with a *p* value <0.05 and |log 2(fold change)| > 0.5 were collected. Moreover, 739 GO entries of HGWD on RA were identified, and 79 pathways were screened based on GO and KEGG analysis. The core target gene of HGWD in RA treatment was JUN. Other key target genes included FOS, CCND1, IL6, E2F2, and ICAM1. It was confirmed that the TNF signaling pathway and IL-17 signaling pathway are important pathways of HGWD in the treatment of RA. The molecular docking results revealed that the top 10 active compounds of HGWD had a strong binding to the target proteins of VCAM1, CTNNB1, and JUN.

**Conclusion:**

HGWD has important active compounds such as quercetin, kaempferol, and beta-sitosterol, which exert its therapeutic effect on multiple targets and multiple pathways.

## 1. Introduction

Rheumatoid arthritis (RA), a common chronic systemic autoimmune disease, is characterized by synovial hyperproliferation and inflammatory/immune cell infiltration [1, 2]. Typical clinical symptoms of RA include tender and swelling of the joints, accompanied by morning stiffness of the affected joints. In severe cases, large joints can be injured leading to joint deformity and loss of function. The current global incidence of RA is 0.24%, and it is expected to increase [3–6]. Besides, RA is ranked 74^th^ as a social burden and 42^nd^ as a disability [3–6]. Current international guidelines for the management of RA recommend the use of disease-modifying antirheumatic drugs (DMARDs), with methotrexate (MTX) as the first-line drug [7, 8]. However, MTX is not an ideal therapeutic agent. It has damaging side effects on the neuronal, gastrointestinal, and immune systems [9]. Therefore, it is imperative to explore safe and effective clinical treatment for RA.

The traditional Chinese medicine (TCM) is based on the theory of syndrome differentiation and has long been established as an effective treatment of RA. Huangqi Guizhi Wuwu Decoction (HGWD), a classical prescription described in *Jingui Yaolue*, has been used over 1000 years in China to treat joint pain. HGWD was formulated by Zhang Zhongjing during the Han dynasty. It is a mixture of five Chinese medicines including *Astragalus membranaceus* (Huangqi), Cassia Twig (Guizhi), *Radix Paeoniae Alba* (Baishao), ginger (Shengjiang), and Chinese date (Dazao). HGWD is considered to have the ability to reinforce Qi and nourish blood, warm and smoothen meridian, and dredge arthritic pain traditionally [10]. In a study of rats with RA, HGWD administration reduced joint inflammation, synovial hyperplasia, and cartilage damage [11]. Previous studies have revealed that HGWD improves the clinical symptoms and signs, as well as laboratory indices, in RA patients [12]. Moreover, a systematic review and meta-analysis showed that when HGWD was combined with Western medicine therapy to treat RA, better efficacy, improved morning stiffness, reduced C-reactive protein, and rheumatoid factor content were achieved compared to Western medicine only [13]. However, the mechanisms of HGWD in RA treatment are not clear, a major limiting factor for its extensive application.

Network pharmacology is partly bioinformatics and was first proposed by Hopkins [14]. It has been successfully used to study complex TCM formulations. This is because it not only combines system network analysis and pharmacology but is also based on the connotation of holistic theory, multicomponents, multitargets, and multipathways of Chinese medicine [15, 16]. Network pharmacology can elucidate the mechanisms of HGWD in the RA treatment at the molecular level via compound-compound network, compound-target network, and target-disease network.

**Figure 1:**
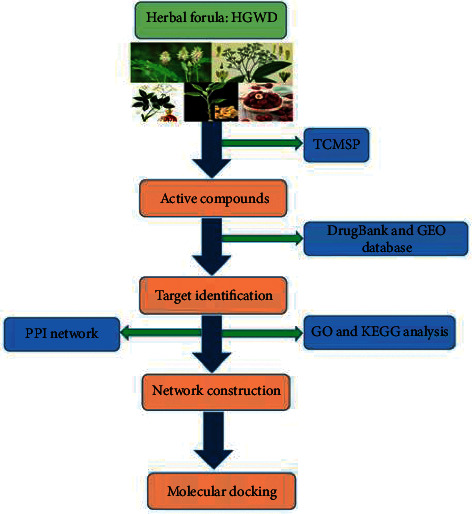
The flow diagram of network pharmacology analysis.

In the present study, network pharmacology was used to establish a compound-target-disease network for exploring the potential HGWD mechanism of action in RA treatment. This study provides a reference for future pharmacological studies and clinical applications. The flow diagram of the network is shown in Figure 1.

## 2. Methods

### 2.1. Collection of Active HGWD Compound Information

Information on the HGWD compounds was retrieved from the Traditional Chinese Medicine Systems Pharmacology Database (TCMSP, http://tcmspw.com/tcmsp.php) [17]. TCMSP is a unique system pharmacology database of Chinese herbal medicines with data on absorption, distribution, metabolism, and excretion (ADME)-related parameters of herbal ingredients as well as the relationships between diseases, targets, ingredients, and drugs [18]. The active compounds of HGWD were primarily screened based on oral bioavailability (OB) and drug-like (DL) properties, the two important indicators for bioinformatics evaluation of ADME characteristics [19]. The OB is a major pharmacokinetic parameter of oral drugs and is the proportion of oral drug dose in the systemic circulation [20]. DL properties are the physical and chemical properties that qualitatively evaluate whether a compound is similar to existing drugs [21]. Subsequently, the compounds with OB ≥ 30% and DL ≥ 0.18 were chosen as the candidate compounds for further analysis.

### 2.2. Identification of HGWD Potential Targets

Target identification is an important aspect of drug exploration [22]. The target candidate compounds were imported into the DrugBank database (https://www.drugbank.ca/) to identify the corresponding potential targets of HGWD [23].

### 2.3. Known RA-Related Targets

The differentially expressed genes in RA were retrieved from the GEO database (https://www.ncbi.nlm.nih.gov; series: GSE21959; samples: there were 36 samples, 18 for healthy individuals and 18 for those with rheumatoid arthritis). The genes with a *p* value <0.05 and |log 2(fold change)| > 0.5 were regarded as differentially expressed genes and RA-related targets.

### 2.4. Network Construction

The compound-target network of HGWD was constructed and visualized via Cytoscape 3.8.0 software [24]. Protein-protein interaction (PPI) networks were constructed to explore the potential mechanisms of HGWD on RA using the plugin BisoGenet of Cytoscape 3.8.0 software [25]. Afterward, the topological importance of each node by calculating degree centrality (DC), betweenness centrality (BC), closeness centrality (CC), eigenvector centrality (EC), local average connectivity-based method (LAC), and network centrality (NC) was evaluated with a Cytoscape plugin CytoNCA. Their definitions and computational formulas have been reported and are the topological importance representative of each node [26].

### 2.5. Gene Ontology and Pathway Enrichment Analysis

Gene ontology (GO) analysis and Kyoto Encyclopedia of Genes and Genomes (KEGG) are important methods that describe the features of candidate targets. The two were performed in R software (Bioconductor, clusterProfiler) with the standard *p* value cutoff of 0.05 and the *q* value of 0.05 [27, 28]. The GO analysis was applied for target protein analysis, and the top 20 functional categories in biological process (BP), cellular component (CC), and molecular function (MF) were chosen. Based on the targets of HGWD in RA treatment, KEGG enrichment analysis was carried out. The top 20 KEGG pathways were selected for plotting a histogram. Meanwhile, the network diagram of the gene pathway was drawn.

### 2.6. Molecular Docking of the Main Active Constituents of HGWD and Core Proteins

The 3D protein structure of the three core proteins corresponding to the core targets, VCAM1, CTNNB1, and JUN, was downloaded from the UniProt database. Subsequently, the structure of the active ingredient in HGWD (the top 10 places in the number of targets) was downloaded from the PubChem database and saved in the PDB format. Using PyMOL software, the three proteins were virtually dehydrated and hydrogenated, the original ligands were extracted in each protein, and then were stored separately. AutoDockTools 1.5.6 was utilized to convert compounds, ligands, and proteins into the “pdbqt” format and to define if the location of each protein or its ligands was the active pocket of the protein. Finally, Vina 1.5.6 was run to assess molecular docking. At a binding energy value <0, the molecular proteins were considered to spontaneously bind and interact with each other. Accordingly, the lower the energy is, the more stable the molecular conformation is.

## 3. Results

### 3.1. Target Screening of HGWD and RA

**Figure 2:**
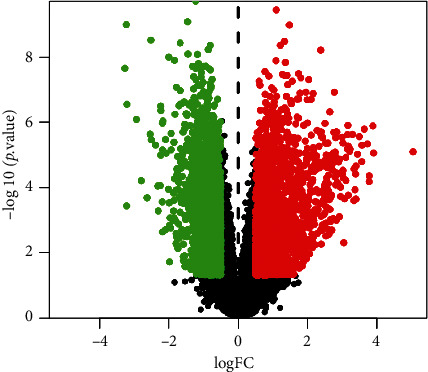
Volcano plot of differentially expressed genes. The red and green dots represent the significant differentially expressed genes.

A total of 790 compounds of the five herb medicines in HGWD were retrieved from the TCMSP database. This included 87 compounds in Huangqi, 220 in Guizhi, 85 in Baishao, 265 in Shengjiang, and 133 in Dazao. Among them, 74 compounds passed OB ≥ 30% and DL ≥ 0.18 filtering. Specifically, the numbers of candidate compounds in Huangqi, Guizhi, Baishao, Shengjiang, and Dazao were 20, 7, 13, 5, and 29, respectively. The candidate compounds in HGWD used for further analysis are shown in Table 1. The DrugBank database retrieval predicted a total of 1006 potential targets. The potential targets linked to Huangqi, Guizhi, Baishao, Shengjiang, and Dazao were 405, 57, 104, 60, and 380, respectively. Moreover, a total of 4570 differentially expressed genes in RA were collected from the GEO database. As shown in Figure 2, a volcano plot was drawn to show the distribution of the differentially expressed genes. The genes are represented by the red and green dots in the plot. We compared the target genes regulated by the active compounds in HGWD, and different genes in RA were compared, obtaining 49 common target genes. These 49 target genes were found to be regulated by 28 active compounds.

### 3.2. Compound-Target Network Analysis

**Figure 3:**
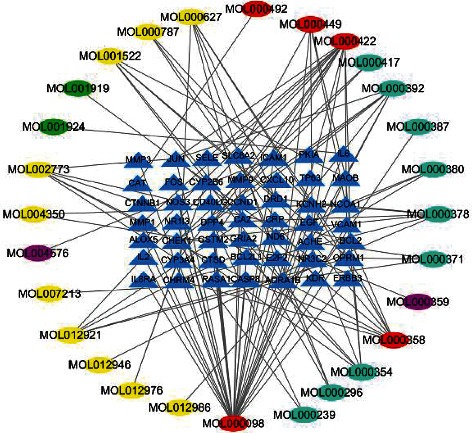
Compound-target network of the HGWD formula. The blue triangles represent the targets, and the ellipses represent active compounds. The green, yellow, amaranth, wathet, and red colors represent compounds from Baishao, Dazao, Guizhi, Huangqi, and multidrug, respectively.

The compound-target network of HGWD was established with the collected compounds and their targets as shown in Figure 3. The network contains 77 nodes (28 compounds in HGWD and 49 compound targets) and 130 edges elucidating the compound-target interactions. Quercetin, kaempferol, and beta-sitosterol acted on 33, 14, and 8 targets, respectively. The OB of quercetin, kaempferol, and beta-sitosterol was 43.43, 41.88, and 36.91%, respectively. Therefore, they are potential key active compounds due to their relative positioning in the network. Besides, it has been revealed that Guizhi, Baishao, and Dazao share a common component (+)-catechin (ID: MOL000492); Shengjiang and Dazao have the same ingredient stigmasterol (ID: MOL000449); Guizhi, Baishao, Shengjiang, and Dazao share a common ingredient beta-sitosterol (ID: MOL000358); Huangqi and Baishao share the same constituent kaempferol (ID: MOL000422); and Huangqi and Dazao share the same component quercetin (ID: MOL000098).

### 3.3. The Candidate Targets for HGWD against RA

**Figure 4:**
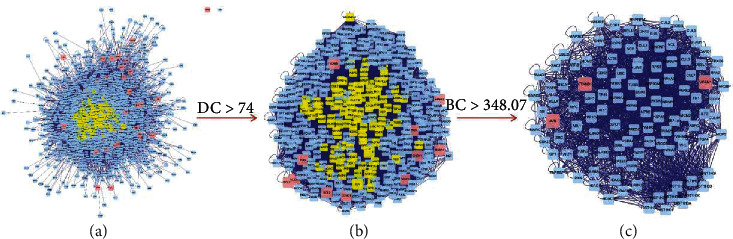
Protein interaction network of the HGWD formula.

To elucidate the mechanism by which HGWD ameliorates RA, the potential target network was merged with the RA-related target network to form a core PPI network of 2284 nodes and 53,119 edges (Figure 4(a)). The previous research showed that the median degree of all nodes was more than 74 degrees which were identified as significant targets [29]. Accordingly, a network of the significant targets for HGWD against RA with 426 nodes and 17,112 edges with a DC ≥ 74 was constructed (Figure 4(b)).

During the second screening, since the number of genes was limited, only BC average value was used. The candidate targets were further identified, and 111 targets with BC ≥ 348.07 (BC average value) were chosen (Figure 4(c)). Eventually, 111 HGWD target genes against RA were identified. VCAM1 (degree: 452), CTNNB1 (degree: 390), and JUN (degree: 274) might be the most important among 111 target genes for HGWD against RA.

### 3.4. GO and KEGG Pathway Enrichment Analysis

**Figure 5:**
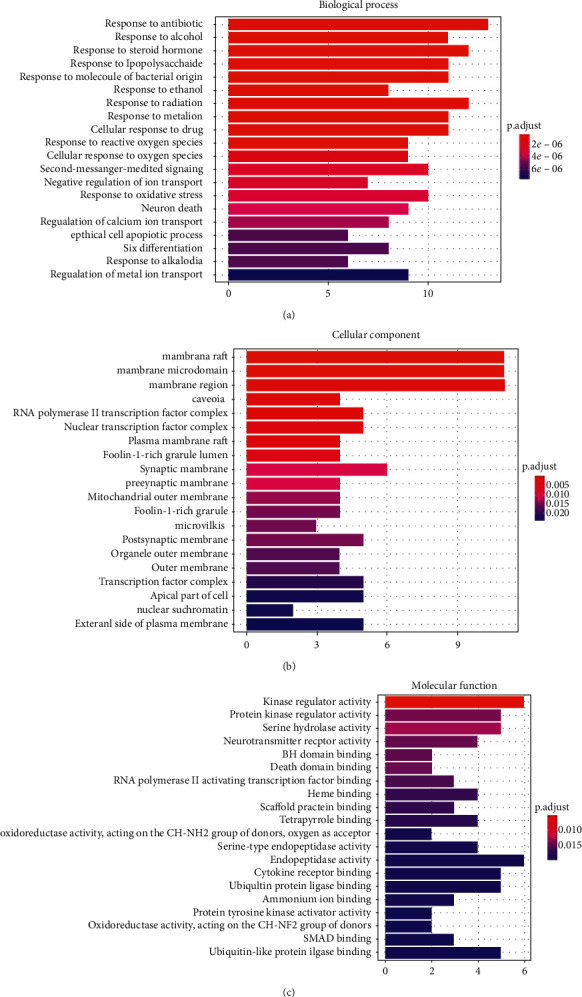
Gene ontology terms of the candidate targets of the HGWD formula against RA. (a) Biological process. (b) Cellular component. (c) Molecular function.

To further confirm the biological responses from RA treatment with HGWD, GO analysis of the 49 RA-related potential therapeutic target genes was performed based on BP, CC, and MF. The analysis results revealed a total of 739 entries. In BP enrichment analysis, 666 entries were obtained including response to antibiotic, response to alcohol, and response to steroid hormone. In CC enrichment analysis, 34 entries involved in membrane raft, membrane microdomain, membrane region, etc. were obtained. The MF enrichment analysis revealed 39 entries, including kinase regulator activity, protein kinase regulator activity, and serine hydrolase activity. The top 20 terms are shown in Figure 5.

**Figure 6:**
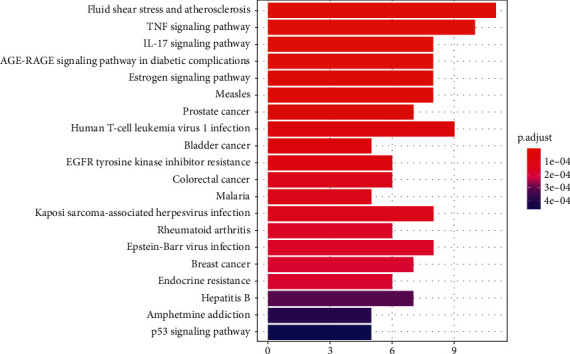
KEGG pathway enrichment of the candidate targets of the HGWD formula against RA.

To further show the biological processes of these targets, the KEGG pathway analysis was performed. The analysis results showed that these targets were enriched in 79 pathways with an adjusted *p* value <0.05. The top 20 KEGG pathways' enrichment analysis is shown in Figure 6. Among these pathways, the top three were fluid shear stress and atherosclerosis, TNF signaling pathway, and IL-17 signaling pathway based on the number of the pathway target genes.

### 3.5. The Gene-Pathway Network

**Figure 7:**
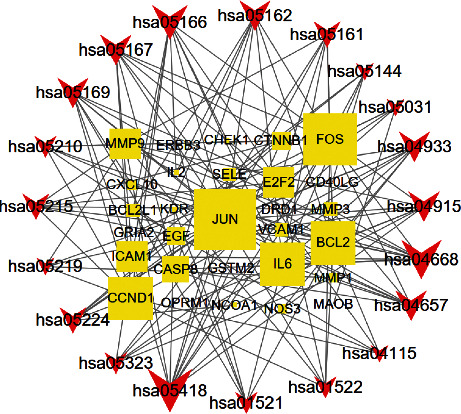
Gene-pathway network of the HGWD formula against RA. The yellow squares represent the target genes, and the red v-shapes represent pathways. The large size represents the larger betweenness centrality.

The gene-pathway network analysis was constructed according to the significantly enriched pathways and genes which regulate these pathways as shown in Figure 7. The diagram shows the network relationship between the top 20 pathways and their regulated target genes. According to the network analysis, JUN had the highest volume, hence the core target gene. Additionally, other genes had a relatively high volume including FOS, CCND1, IL6, E2F2, and ICAM1. These genes are potential key target genes involved in the HGWD treatment of RA.

### 3.6. The Molecular Docking of the Main Active Constituents of HGWD and Core Proteins

**Figure 8:**
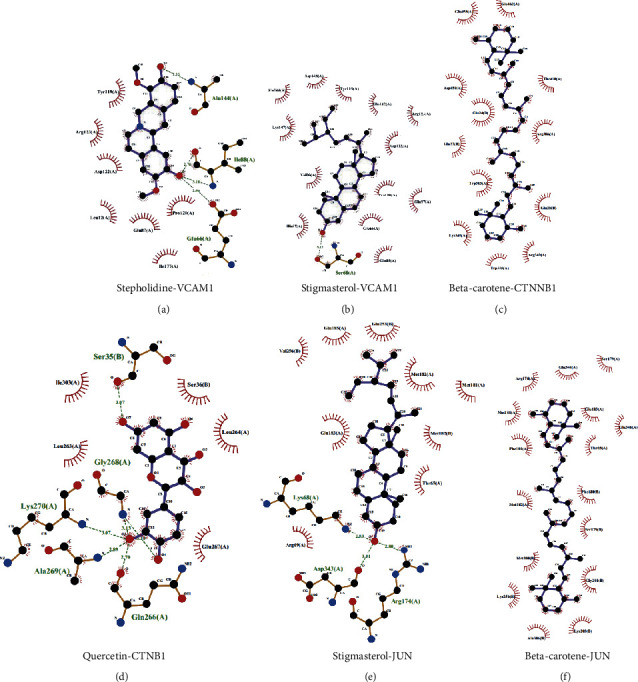
Molecular docking diagram. (a) Stepholidine-VCAM1. (b) Stigmasterol-VCAM1. (c) Beta-carotene-CTNNB1. (d) Quercetin-CTNB1. (e) Stigmasterol-JUN. (f) Beta-carotene-JUN.

Normally, high connectivity compounds are associated with more targets. According to the number of related targets, the top 10 active compounds in HGWD with high connectivity in the compound-target network were selected for molecular docking (Table 2, binding energy of the main potential active ingredients in HGWD). Based on the binding energy value, the lower the binding energy value, the stronger the binding to the target protein. Among them, stigmasterol and stepholidine had the strongest binding force with VCAM1 (PDB: 1ij9), beta-carotene and quercetin had the strongest binding force with CTNNB1 (PDB: 1jdh), and beta-carotene and stigmasterol had the strongest binding force with JUN (PDB: 2g01). The corresponding molecular docking diagram of stepholidine, stigmasterol, and VCAM1, beta-carotene, quercetin and CTNNB1, and beta-carotene, stigmasterol, and JUN is illustrated in Figure 8.

## 4. Discussion

TCM, characterized by multicompound and multitarget medicines, cures diseases via multiple targets, multiple pathways, and multiple links. Due to the complex chemical ingredients of TCM, conventional research methods such as clinical and pharmacological research are not capable of fully elucidating the mechanism of action of TCM. Fortunately, network pharmacology provides a solution to this challenge since it is suitable for multicompound and multitarget research. In the present study, the mechanism of action of HGWD on RA was explored via network pharmacology methods. This provided a clear direction for further basic and clinical research.

Modern pharmacology has shown that HGWD has a specific therapeutic effect on RA, where it elicits obvious anti-inflammatory and analgesic effects [30]. Specifically, Huangqi has an anti-inflammatory effect and improves the immunity [31]. The volatile oil of Guizhi has good anti-inflammatory, immunological, and chondrocyte proliferation effects [32]. The total glycosides, the main components of Baishao, have anti-inflammatory, analgesic, and autoimmunological effects [33]. Shengjiang resists oxidation, scavenges free radicals, and relieves pain [34]. The polysaccharide of Dazao can significantly inhibit proinflammatory cytokines, such as IL-6 and TNF, with anti-inflammatory activity [35]. It is suggested that this prescription has anti-inflammatory, antioxidation, analgesic, and autoimmune reaction pharmacological effects, thus providing specific pharmacological basis for its clinical function in the treatment of RA.

In this study, the compound-target network of HGWD was constructed using 28 compounds and their 49 targets. The network diagram demonstrated that most of the HGWD compounds affected multiple targets. For instance, quercetin, kaempferol, and beta-sitosterol acted on 33, 14, and 8 targets, respectively. Therefore, they were the likely key active compounds for HGWD. Quercetin is a common active component of Huangqi and Dazao. Kaempferol is a common active component of Huangqi and Baishao. Beta-sitosterol is a common active component of Guizhi, Baishao, Shengjiang, and Dazao. These drugs have been mainly used together in Chinese medical history. It indicates that the compatibility between these drugs has a synergistic effect and increases their efficacy.

Quercetin is a flavonol that has unique therapeutic biological properties including anticarcinogenic, anti-inflammatory, antioxidant, antiviral, and psychostimulant activities [36, 37]. Kaempferol, a representative flavonoid, has been known to exert a range of pharmacological actions including the mediation of antioxidant, antimicrobial, and anti-inflammatory effects [38, 39]. Kaempferol is a potent immunosuppressant that reduces the harmful immune responses including autoimmunity and chronic inflammation [40]. Beta-sitosterol, a main constituent of plants and vegetables, has versatile activities that impact cell activities including anti-inflammatory effect [41, 42]. In this study, quercetin, kaempferol, and beta-sitosterol regulated most RA-related targets and exhibited anti-inflammatory effects. Besides, they have a high oral bioavailability. Therefore, they were identified as the representative compounds of HGWD.

The PPI networks of HGWD targets and RA-related targets were constructed and merged to identify the candidate targets of HGWD against RA. To accurately obtain the targets, two parameters including DC and BC were set to construct a new network. Through PPI analysis, VCAM1, CTNNB1, and JUN were established as the important targets in RA treatment. VCAM-1 is a glycoprotein expressed in vascular endothelial cells, whose serum is positively correlated with RA [43]. Studies have found that [44] TNF-*α* upregulates the expression of VCAM-1 in the endothelial cell membrane at the small joint synovium. VCAM-1 binds to *α*4*β*1 on the surface of free immune cells in the blood vessels, inducing the migration of immune cells to inflammatory joints thus expanding the inflammatory response cascade and aggravating small joint injury. Proliferation and invasion of fibroblast synovial cells are important mechanisms that result in the thickened synovial lining, increased secretion of inflammatory cytokines, and cartilage and bone injury [45]. Studies have shown that inhibition of CTNNB1 transcription reduces the proliferation of fibroblast synovial cells and the levels of proinflammatory cytokines such as interleukin-6 (IL-6), IL-10, and TNF-*α* [46]. Elsewhere, it has been shown that CD44 can activate the transcription factor AP-1 (the whole is a protein) expressed by the gene JUN, thus promoting the activation of T cells and aggravating RA synovitis [47].

The targets of HGWD against RA were enriched in BP, CC, and MF through GO analysis. Results suggested that HGWD mainly regulated response to antibiotics, response to steroid hormones, and response to radiation. Furthermore, it was shown to affect some cellular components and molecular functions including membrane raft, membrane microdomain, membrane region, and kinase regulator activity. In the present study, 79 KEGG pathways including TNF signaling pathway and IL-17 signaling pathway were significantly enriched. IL-17 and TNF-*α* are classic proinflammatory cytokines. IL-17 is mainly secreted by helper T-cells type 17 and has strong proinflammatory effects. Analysis of synovial fluid in patients with RA found that IL-17 content was significantly increased and positively correlated with Disease Activity Score-28 (DAS28) [48]. This was a confirmation that IL-17 is involved in the occurrence of RA. Fischer et al. [49] found that, after IL-17 treatment of fibroblast synovial cells, the expression of proinflammatory cytokines such as IL-6, IL-8, and granulocyte-macrophage colony-stimulating factor (GM-CSF) increases, aggravating the inflammatory damage of fibroblast synovial cells. Besides, IL-17 can also induce joint bone and cartilage damages [50]. It has been revealed that IL-17 upregulates the levels of nuclear factor-kappa B (NF-*κ*B) receptor activator ligand RANKL. Consequently, this increases the content of matrix metalloproteinases, possibly promoting cartilage degradation, inhibiting chondroprogenitor cell formation, and stimulating osteoclast bone damaging [51–53].

TNF-*α* plays an important role in the development of RA. On the one hand, it can bind to the TNFR1 receptor on fibroblast synovial cells, promoting the release of inflammatory cytokines such as IL-1, IL-6, and IL-8 and aggravating the damage of the articular cartilage and bone [54]. On the other hand, TNF-*α* can stimulate immune cells in blood to enter the joint through VCAM-1, aggravating the joint injury [44]. Besides, the other pathways in the first 20 pathways have not been reported to be related to RA, providing new ideas and clues for future research.

The gene-pathway network was constructed to explore the main target genes of HGWD against RA. The results revealed that JUN, FOS, CCND1, IL6, E2F2, and ICAM1 are important target genes. A study found that the ERK-JNK-P38 signaling pathways in autoimmune disease are activated, leading to high levels of downstream JUN and Fos protein. Subsequently, the two combine to form dimer transcription factor-activating protein 1 (AP-1), which is involved in the occurrence and development of RA. It regulates the transformation of initial T cells into effector T cells, hence regulating the immune response and inflammatory process [55–58]. Inflammatory response runs throughout RA. The proinflammatory cytokines such as TNF-*α*, IL-6, and IL-17 play a crucial role in synovial fibroblast inflammation. Studies have demonstrated that TNF-*α* upregulates the E2F2 expression by activating the nuclear transcription factor NF-*κ*B pathway. This enhances synovial cell proliferation and synovial tissue thickening leading to joint injury [59]. The serum level of intercellular adhesion molecule-1 (ICAM-1) is high in RA patients. It has been found that ICAM-1 induces inflammatory cell aggregation and promotes synovial cell inflammation once it combines with ligand lymphocyte function antigen-1 (LFA-1) [60]. Through the molecular docking analysis of the effects of the chief active components in HGWD and the core target proteins of RA, the molecular mechanism of the treatment of RA by the active components of TCM can be predicted. This is of great reference significance for subsequent research and development of targeted drugs. The results showed that the main compounds in HGWD have a strong binding force to the core proteins VCAM1, CTNNB1, and JUN. Among them, stigmasterol and stepholidine closely link to VCAM1 through hydrogen bonds and hydrophobic forces. Beta-carotene and quercetin closely link to CTNNB1 through hydrogen bonds and hydrophobic interactions. Stigmasterol closely links to JUN through hydrogen bonds and hydrophobic interactions. Beta-carotene closely links to JUN only through hydrophobic interactions. Based on this, it can be concluded that stigmasterol, stepholidine, beta-carotene, and quercetin are the key compounds in the treatment of RA.

## 5. Conclusion

Our study systematically elucidated the mechanisms of action and molecular targets of HGWD against RA via the network pharmacology approach. Quercetin, kaempferol, and beta-sitosterol regulated most of the targets related to RA. The HGWD can regulate gene function through their related pathways including TNF signaling pathway and IL-17 signaling pathway. The key target genes in the gene-pathway network of HGWD against RA were JUN, FOS, CCND1, IL6, E2F2, and ICAM1. Furthermore, according to molecular docking analysis, important compounds such as stepholidine, stigmasterol, beta-carotene, quercetin, and the core protein CTNNB1, VCAM1, and JUN all have good binding ability. The network pharmacology is a promising suitable approach for the study of TCM formulations.

## Figures and Tables

**Table 1:** Basic information of the active compounds in the HGWD formula.

Molecule ID	Name	OB	DL	Source
MOL000211	Mairin	55.38	0.78	Huangqi, Baishao, Dazao
MOL000239	Jaranol	50.83	0.29	Huangqi
MOL000296	Hederagenin	36.91	0.75	Huangqi
MOL000033	(3S,8S,9S,10R,13R,14S,17R)-10,13-Dimethyl-17-[(2R,5S)-5-propan-2-yloctan-2-yl]-2,3,4,7,8,9,11,12,14,15,16,17-dodecahydro-1H-cyclopenta[a]phenanthren-3-ol	36.23	0.78	Huangqi
MOL000354	Isorhamnetin	49.6	0.31	Huangqi
MOL000371	3,9-Di-O-methylnissolin	53.74	0.48	Huangqi
MOL000374	5′-Hydroxyiso-muronulatol-2′,5′-di-O-glucoside	41.72	0.69	Huangqi
MOL000378	7-O-Methylisomucronulatol	74.69	0.3	Huangqi
MOL000379	9,10-Dimethoxypterocarpan-3-O-*β*-D-glucoside	36.74	0.92	Huangqi
MOL000380	(6aR,11aR)-9,10-Dimethoxy-6a,11a-dihydro-6H-benzofuro[3,2-c]chromen-3-ol	64.26	0.42	Huangqi
MOL000387	Bifendate	31.1	0.67	Huangqi
MOL000392	Formononetin	69.67	0.21	Huangqi
MOL000398	Isoflavanone	109.99	0.3	Huangqi
MOL000417	Calycosin	47.75	0.24	Huangqi
MOL000422	Kaempferol	41.88	0.24	Huangqi, Baishao
MOL000433	FA	68.96	0.71	Huangqi
MOL000438	(3R)-3-(2-Hydroxy-3,4-dimethoxyphenyl)chroman-7-ol	67.67	0.26	Huangqi
MOL000439	Isomucronulatol-7,2′-di-O-glucosiole	49.28	0.62	Huangqi
MOL000442	1,7-Dihydroxy-3,9-dimethoxy pterocarpene	39.05	0.48	Huangqi
MOL000098	Quercetin	46.43	0.28	Huangqi, Dazao
MOL001736	(-)-Taxifolin	60.51	0.27	Guizhi
MOL000358	Beta-sitosterol	36.91	0.75	Guizhi、Baishao、Shengjiang、Dazao
MOL000359	Sitosterol	36.91	0.75	Guizhi, Baishao
MOL000492	(+)-Catechin	54.83	0.24	Guizhi, Baishao, Dazao
MOL000073	ent-Epicatechin	48.96	0.24	Guizhi
MOL004576	Taxifolin	57.84	0.27	Guizhi
MOL011169	Peroxyergosterol	44.39	0.82	Guizhi
MOL001928	Albiflorin_qt	66.64	0.33	Baishao
MOL001918	Paeoniflorgenone	87.59	0.37	Baishao
MOL001910	11alpha,12alpha-Epoxy-3beta-23-dihydroxy-30-norolean-20-en-28,12beta-olide	64.77	0.38	Baishao
MOL001925	Paeoniflorin_qt	68.18	0.4	Baishao
MOL001919	(3S,5R,8R,9R,10S,14S)-3,17-Dihydroxy-4,4,8,10,14-pentamethyl-2,3,5,6,7,9-hexahydro-1H-cyclopenta[a]phenanthrene-15,16-dione	43.56	0.53	Baishao
MOL001930	Benzoyl paeoniflorin	31.27	0.75	Baishao
MOL001924	Paeoniflorin	53.87	0.79	Baishao
MOL001921	Lactiflorin	49.12	0.8	Baishao
MOL006129	6-Methylgingediacetate2	48.73	0.32	Shengjiang
MOL000449	Stigmasterol	43.83	0.76	Shengjiang, Dazao
MOL001771	Poriferast-5-en-3beta-ol	36.91	0.75	Shengjiang
MOL008698	Dihydrocapsaicin	47.07	0.19	Shengjiang
MOL012921	Stepharine	31.55	0.33	Dazao
MOL012940	Spiradine A	113.52	0.61	Dazao
MOL012946	Zizyphus saponin I_qt	32.69	0.62	Dazao
MOL012961	Jujuboside A_qt	36.67	0.62	Dazao
MOL012976	Coumestrol	32.49	0.34	Dazao
MOL012980	Daechuine S6	46.48	0.79	Dazao
MOL012981	Daechuine S7	44.82	0.83	Dazao
MOL012986	Jujubasaponin V_qt	36.99	0.63	Dazao
MOL012989	Jujuboside C_qt	40.26	0.62	Dazao
MOL012992	Mauritine D	89.13	0.45	Dazao
MOL001454	Berberine	36.86	0.78	Dazao
MOL001522	(S)-Coclaurine	42.35	0.24	Dazao
MOL003410	Ziziphin_qt	66.95	0.62	Dazao
MOL004350	Ruvoside_qt	36.12	0.76	Dazao
MOL005360	Malkangunin	57.71	0.63	Dazao
MOL000627	Stepholidine	33.11	0.54	Dazao
MOL007213	Nuciferin	34.43	0.4	Dazao
MOL000783	Protoporphyrin	30.86	0.56	Dazao
MOL000787	Fumarine	59.26	0.83	Dazao
MOL008034	21302-79-4	73.52	0.77	Dazao
MOL008647	Moupinamide	86.71	0.26	Dazao
MOL002773	Beta-carotene	37.18	0.58	Dazao
MOL000096	(-)-Catechin	49.68	0.24	Dazao
MOL013357	(3S,6R,8S,9S,10R,13R,14S,17R)-17-[(1R,4R)-4-Ethyl-1,5-dimethylhexyl]-10,13-dimethyl-2,3,6,7,8,9,11,12,14,15,16,17-dodecahydro-1H-cyclopenta[a]phenanthrene-3,6-diol	34.37	0.78	Dazao

*Note.* OB, oral bioavailability; DL, drug-likeness.

**Table 2:** The binding energy of the main potential active ingredients in the HGWD formula.

Compound	Molecular formula	Binding energy (VCAM1)	Binding energy (CTNNB1)	Binding energy (JUN)
Quercetin	C_15_H_10_O_7_	−7.1	−7.3	−7.3
Kaempferol	C_15_H_10_O_6_	−7.1	−6.5	−7.4
Beta-sitosterol	C_29_H_50_O	−7.3	−7.1	−7.8
Formononetin	C_16_H_12_O_4_	−7	−6.3	−6.9
7-O-Methylisomucronulatol	C_18_H_20_O_5_	−6.7	−6	−6.9
Isorhamnetin	C_16_H_12_O_7_	−6.9	−6.5	−7.5
Beta-carotene	C_40_H_56_	−6.5	−7.9	−8.1
Stepholidine	C_19_H_21_NO_4_	−7.5	−6.7	−6.9
(S)-Coclaurine	C_17_H_19_NO_3_	−7.1	−6.3	−7.1
Stigmasterol	C_29_H_48_O	−7.6	−7	−8.1

## Data Availability

The data supporting the findings of this study are available from the corresponding author upon request.

## Abbreviations

RA: Rheumatoid arthritis
DMARDs: Disease-modifying antirheumatic drugs
MTX: Methotrexate
TCM: Traditional Chinese medicine
HGWD: Huangqi Guizhi Wuwu Decoction
TCMSP: Traditional Chinese Medicine Systems Pharmacology Database
ADME: Absorption, distribution, metabolism, and excretion
OB: Oral bioavailability
DL: Drug-like properties
PPI: Protein-protein interaction
DC: Degree centrality
BC: Betweenness centrality
CC: Closeness centrality
EC: Eigenvector centrality
LAC: Local average connectivity-based method
NC: Network centrality
GO: Gene ontology
KEGG: Kyoto Encyclopedia of Genes and Genomes
BP: Biological process
CC: Cellular component
MF: Molecular function
IL-6: Interleukin-6
DAS28: Disease Activity Score-28
GM-CSF: Granulocyte-macrophage colony-stimulating factor
NF-*κ*B: Nuclear factor-kappa B
AP-1: Activating protein-1
ICAM-1: Intercellular adhesion molecule-1
LFA-1: Lymphocyte function antigen-1.
